# Supplementary material for: On the proportional abundance of species: Integrating population genetics and community ecology
Source: Sci Rep. 2017 Dec 1;7:16815. doi: 10.1038/s41598-017-17070-1 (PMC5711905; doi:10.1038/s41598-017-17070-1)
Supplement: Supplementary file 1 — Supplementary Information [file 41598_2017_17070_MOESM1_ESM.pdf]

SUPPLEMENTARY INFORMATION
On the proportional abundance of species: Integrating population genetics and community ecology

Pablo A. Marquet[1,2,3,4,5,*], Guillermo Espinoza[1,6], Sebastian R. Abades[7], Angela Ganz[6], and Rolando Rebolledo[6,8]

[1]Departamento de Ecología, Facultad de Ciencias Biológicas, Pontificia Universidad Católica de Chile, Alameda 340 C.P. 6513677, Santiago, Chile
[2]Instituto de Ecología y Biodiversidad (IEB), Las Palmeras 3425, Santiago, Chile
[3]Instituto de Sistemas Complejos de Valparaíso (ISCV), ArtillerÃa 470, Cerro Artillerí, Valparaíso, Chile
[4]Laboratorio Internacional en Cambio Global (LINCGlobal) and Centro de Cambio Global (PUCGlobal), Pontificia Universidad Católica de Chile, Alameda 340 C.P. 6513677, Santiago, Chile
[5]The Santa Fe Institute, 1399 Hyde Park Road, Santa Fe, NM 87501, USA
[6]Centro de Análisis Estocástico y Aplicaciones, Facultad de Ingeniería and Facultad de Matemáticas, Pontificia Universidad Católica de Chile, Casilla 306, Santiago 22, Chile
[7]GEMA Center for Genomics, Ecology & Environment, Universidad Mayor, Camino La Pirámide 5750 Huechuraba, Chile
[8]Centro de Investigación y Modelamiento de Fenómenos Aleatorios (CIMFAV), Universidad de Valparaíso, Chile
[*]Correspondence and requests for materials should be addressed to P.A.M.(email:pmarquet@bio.puc.cl)

We start by defining a general population model and its diffusion approximation. To do so, we envision the situation where an observer is able to characterize the state of an ecological system at a given scale in time and space (i.e. the focal system) by measuring several parameters, in our case the important ones are:

- S or the number of species in the focal system.
- J or total number of individuals in the focal system.
- $N_J(t)$ is the number of individuals of a given species during the time interval $[0,t]$, inside a focal community of size $\leq J$.

Let us now define the proportion $X_J(t) = \dfrac{N_J(t)}{J}$ that corresponds to a (random) proportional abundance during $]0,t]$. We are interested in the behavior of this proportion when the size of the population J increases to infinity to find the law (or the *state* of the open system), when the proportions will become probabilities. This requires to rescale time t by J. Thus, for each total number of individual J we define the rescaled proportional abundance process $Z_J(t) = \dfrac{N_J(Jt)}{J} = X_J(Jt)$. According to the Neutral Theory all species are indistinguishable, so that the expected value of $Z_J(t)$, is $\mathbb{E}(Z_J(t)) = \mathbb{E}(\dfrac{N_J(Jt)}{J})$, and represents the proportional abundance of any species.

The dynamics of the population of a given species in the focal system will be governed by generalized birth and death events (including speciation, immigration and emigration) described by two rates b_J and d_J (birth and death of individuals), while the interaction with the environment (unobserved dynamics) is driven by a noise (a martingale). So, let $J \geq 1$ and assume that the process $(N_J(t),\ t \geq 0)$ is a birth and death process taking values in $\{1, \ldots, J\}$.

The transition probabilities are given by

$$Q_J(x,y) = \begin{cases} B_J(x) \text{ if } y = x+1,\ 0 \leq x \leq J-1\ , \\ 1 - (B_J(x) + D_J(x)) \text{ if } y = x,\ 0 \leq x \leq J\ , \\ D_J(x) \text{ if } y = x-1,\ 1 \leq x \leq J\ , \\ 0 \text{ otherwise.} \end{cases} \tag{S1}$$

$X_J(t) = N_J(t)/J$ is again a jump Markov process with states in $\{0, \frac{1}{J}, \frac{2}{J}, \ldots, 1\} \subset [0,1]$, and we abuse the language by keeping the same notations for the transition kernel: $Q_J(x,y) = B_J(x)$ if $y = x + \frac{1}{J}$, $x \in \{0, \frac{1}{J}, \ldots, 1 - \frac{1}{J}\}$, and so on. The dynamics of this process X_J is typically open: it concerns a main-system part defined by an observable like the evolution rate of X_J; and a noisy part which represents the interaction of this system with the environment.

From the mathematical point of view, X_J can be decomposed as follows:

$$X_J(t) = X_J(0) + \widetilde{X_J}(t) + M_J(t), \tag{S2}$$

where $\widetilde{X_J}$ is the observable process (*predictable*, in mathematical terms), and M_J is the noise process (a *martingale*).

The process $\widetilde{X_J}$ is easily computed by means of the Markov property of X_J (see (*1*)). That requires to introduce the filtration or history induced by X_J, given roughly by $\mathcal{F}_t^J$ obtained from $\sigma(X_J(s); 0 \leq s \leq t)$ by customary Dellacherie procedure for all $t \geq 0$. We assume further that we choose a nice version of X_J, that is right-hand continuous with left-hand limits. As a result, given any measurable function f defined on $[0,1]$, the predictable compensator $\widetilde{f \circ X_J}(t)$ of $f \circ X_J(t) = f(X_J(t))$ is given by

$$\widetilde{f \circ X_J}(t) = \int_0^t B_J(X_J(s-)) \left(f(X_J(s-) + \frac{1}{J}) - f(X_J(s-)) \right) ds + \int_0^t D_J(X_J(s-)) \left(f(X_J(s-) - \frac{1}{J}) - f(X_J(s-)) \right) ds. \tag{S3}$$

So that, if one applies the above formula to the identity function in $[0,1]$, one obtains:

$$\widetilde{X_J}(t) = \frac{1}{J} \int_0^t (B_J(X_J(s-)) - D_J(X_J(s-)))ds. \tag{S4}$$

This gives the main dynamics for the proportion of living individuals in the population of size J.

The noise is (trivially) given by

$$M_J(t) = X_J(t) - \frac{1}{J} \int_0^t (B_J(X_J(s-)) - D_J(X_J(s-)))ds. \tag{S5}$$

However, the important characteristics of this noise is provided by its "energy dissipation", which is the increasing process $\langle M_J, M_J \rangle$ such that $M_J^2 - \langle M_J, M_J \rangle$ is a martingale (or the predictable compensator of the square of the noise, which is an observable quantity). Using again the Markov property one finds

$$\langle M_J, M_J \rangle(t) = \frac{1}{J^2} \int_0^t (B_J(X_J(s-)) + D_J(X_J(s-)))ds. \tag{S6}$$

The two processes (S4) and (S6) are essential to understand the approximation of the dynamics by a diffusion when $J \to \infty$ and one considers a large time scale (Jt instead of t).

Let define $Z_J(t) = X_J(Jt)$. Therefore, after an elementary change of variables ($u = s/J$) in (S4), we obtain the predictable compensator of Z_J as

$$\begin{aligned} \widetilde{Z_J}(t) &= \frac{1}{J} \int_0^{Jt} (B_J(X_J(s-)) - D_J(X_J(s-)))ds \\ &= \int_0^t (B_J(Z_J(u-)) - D_J(Z_J(u-)))du. \end{aligned}$$

Analogously, in (S6) the new time scale yields

$$\langle M_J, M_J\rangle(Jt) = \frac{1}{J}\int_0^t (B_J(Z_J(u-)) + D_J(Z_J(u-)))du.$$

Theorem 1 *Define $Z_J(t) = X_J(Jt)$, for all $J \geq 1$ and $t \geq 0$. Assume $Z_J(0) = z \in [0,1]$ fixed, and that there exists two continuous functions $\beta, \sigma : [0,1] \to \mathbb{R}$, with $\sigma(x) > 0$, for all $x \in]0,1[$, $\beta \in C^1(]0,1[)$, $\sigma \in C^2(]0,1[)$, such that they satisfy in addition the two following hypotheses:*

(H1) For all $T > 0$, $\sup_{t\in[0,T]} |(B_J(Z_J(t-)) - D_J(Z_J(t-))) - \beta(Z_J(t-))| \to 0$ in probability;

(H2) For all $T > 0$, $\sup_{t\in[0,T]} |\frac{1}{J}(B_J(Z_J(t-)) + D_J(Z_J(t-))) - \sigma^2(Z_J(t-))| \to 0$ in probability, as $J \to \infty$.

Then, the process Z_J converges in distribution towards a diffusion process Z which can be represented as

$$Z(t) = Z(0) + \int_0^t \beta(Z(s))ds + \int_0^t \sigma(Z(s))dW_s,\ (t \geq 0). \tag{S7}$$

Moreover, $Z(t) \in [0,1]$ with probability 1 for all $t \geq 0$. Z is a Feller process and its semigroup $(T_t)_{t\geq 0}$ acting on $C([0,1])$ has a generator L given by

$$Lf(x) = \frac{1}{2}\sigma^2(x)\frac{d^2}{dx^2}f(x) + \beta(x)\frac{d}{dx}f(x),\ (x \in \mathbb{R}), \tag{S8}$$

for any $f \in C^2(]0,1[) \cap C([0,1])$ such that $f(0) = f(1) = 0$. As a result, the dual semigroup $(T_t^*)_{t\geq 0}$ leaves the space $L^1([0,1])$ invariant, so that, in particular, given any probability density ρ on $[0,1]$, its evolution $\rho_t = T_t^*\rho$ satisfies the Chapman-Kolmogorov (or Master Equation),

$$\frac{\partial \rho_t(x)}{\partial t} = L^*\rho_t(x) = \frac{1}{2}\frac{d^2}{dx^2}\left(\sigma^2(x)\rho_t(x)\right) - \frac{d}{dx}\left(\beta(x)\rho_t(x)\right). \tag{S9}$$

Proof. This theorem is a direct consequence of Proposition III.2.4, pages 92-93 in (*2*) (see also a more general result in (*3*)).

One notes first that the process Z_J with states in $[0,1]$ almost surely, has vanishing jumps if $J \to \infty$, since $\sup_t |\Delta Z_J(t)| \leq 1/J$. Thus the first hypothesis in (*2*) Proposition III.2.4, is satisfied.

In addition, given $T > 0$, it holds that

$$\sup_{t\leq T} |\int_0^t (B_J(Z_J(s-)) - D_J(Z_J(s-)))ds - \int_0^t \beta(Z_J(s))ds|$$
$$\leq T \sup_{t\in[0,T]} |(B_J(Z_J(t-)) - D_J(Z_J(t-))) - \beta(Z_J(t-))|.$$

Similarly,

$$\sup_{t\le T}|\frac{1}{J}\int_0^t (B_J(Z_J(s-)) + D_J(Z_J(s-)))ds - \int_0^t \sigma^2(Z_J(s))ds|$$

$$\le T \sup_{t\in[0,T]} |\frac{1}{J}(B_J(Z_J(t-)) + D_J(Z_J(t-))) - \sigma^2(Z_J(t-))|$$

So that, in both previous inequalities the left-hand terms converge to 0 in probability as $J\to\infty$ due to (H1) and (H2).

Moreover, the hypotheses on β and σ imply that there is a unique solution in distribution to the equation (S7) (see for instance (*4*), Corollary 4.29 and Theorem 5.7). As a result, Proposition III.2.4 in (*2*) fully applies. So that, the convergence to the diffusion Z is proved. Moreover, since $\mathbb{P}(Z_J(t)\in[0,1]) = 1$, the convergence in distribution implies that $1 = \limsup \mathbb{P}(Z_J(t)\in[0,1]) \le \mathbb{P}(Z(t)\in[0,1]) \le 1$, thus $Z(t)\in[0,1]$ for all $t\ge 0$, almost surely.

Finally, the coefficients β and σ of the diffusion are bounded, with bounded derivatives, so that, the generator L applies each function of its core $C_c^2(]0,1[)$ into an element of the Banach space $C([0,1])$. Therefore, by a density argument, T_t maps $C([0,1])$ into itself and it is norm-continuous. As a result, the semigroup is of Feller type. Any integrable function of class $C^2(]0,1[)$ is transformed by L^* into an element of $L^1([0,1])$, by a density argument again, $T_t^*(L^1([0,1]))\subset L^1([0,1])$, for all $t\ge 0$, finishing the proof. □

It is worth noticing that the convergence in distribution mentioned in the above theorem, means the convergence of the sequence of laws of the processes on the space of their trajectories. As a result, any continuous functional $F(Z_J)$ of the trajectory of Z_J converges in distribution to $F(Z)$.

Corollary 1 *Consider the sequences B_J and D_J given by equations (5) and (6) in the main text, where the functions $b_J, d_J \in C^1(]0,1[)$ and $c_J\in C^2(]0.1[)$ satisfy equations (7) and (8) in the main text.*

Then, Z_J converges in distribution to a diffusion Z represented as

$$Z(t) = z + \int_0^t (b(Z(s) - d(Z(s))\,ds + \int_0^t \sqrt{2c(Z(s))}dW_s. \tag{S10}$$

Proof. Define $\beta(x) = b(x) - d(x)$, where b and d are given by (7) in the main text. Similarly, define $\sigma^2(x) = 2c(x)$, where c is obtained from (8) in the main text. A simple computation yields,

$$B_J(x) - D_J(x) = \beta(x),$$

for all $x\in[0,1]$, so that (H1) is trivially satisfied. Moreover,

$$\frac{B_J(x)+D_J(x)}{J} = \frac{1}{J}\left(b_J(x) + d_J(x) + 2c_J(x)\right).$$

Since b_J and d_J are bounded, $\lim_J \frac{1}{J}(b_J(x) + d_J(x)) = 0$. And equation (8) in the main text implies that

$$\frac{B_J(x) + D_J(x)}{J} \to \sigma^2(x) = 2c(x),$$

as $J \to \infty$, uniformly in $x \in [0, 1]$. This implies in particular (H2) and the proof is complete. □

It is worth noticing that the distribution P_t of $Z(t)$ represents the **state** of the open ecological system at time t. This state has a density ρ_t, that is $P_t(dx) = \rho_t(x)dx$, and it can be obtained from the process $Z(t)$ as follows: $P_t(Z(t) \in]a, b])$ is the limit of the frequency of trajectories of the process $Z(t)$ visiting the interval $]a, b]$. So that, these frequencies can be obtained by simulating the solutions to (S10).

Derivation of the Beta distribution

The invariant density distribution of $Z(t)$ is the solution of the equation (11) in the main text. The choice of b, d, c according to equations (12), (13), (14) in the main text

yields

$$\gamma \frac{\partial^2}{\partial x^2}\left(x(1-x)\rho_\infty(x)\right) - \frac{\partial}{\partial x}\left((b_0 - d_0) + (b_1 - d_1)x\rho_\infty(x)\right) = 0.$$

Noticing that $b_0 - d_0 = \alpha\gamma$ and $b_1 - d_1 = (\beta - \alpha)\gamma$, the above equation is equivalent to

$$\frac{\partial^2}{\partial x^2}\left(x(1-x)\rho_\infty(x)\right) - \frac{\partial}{\partial x}\left(\alpha + ((\beta - \alpha)x\rho_\infty(x)\right) = 0. \qquad \text{(S11)}$$

A straightforward computation shows that any function of the form

$$x \mapsto Cx^{\alpha-1}(1-x)^{\beta-1}$$

solves (S11). So that, choosing $C = 1/B(\alpha, \beta)$ (normalization constant) one obtains the unique solution $\rho_\infty(x)$ of (S11) which is a probability density on the real line.

In particular, the choice of coefficients (21), (22), (23) (see main text), with $p = 1/S$, leads to

$$\rho_\infty(x) = \frac{1}{B(\alpha, \alpha(S-1))} x^{\alpha-1}(1-x)^{\alpha(S-1)-1}, \qquad \text{(S12)}$$

where $\alpha = \frac{m}{S\lambda(1-m)}$ and $B(\alpha, \alpha(S-1)) = \int_0^1 x^{\alpha-1}(1-x)^{\alpha(S-1)-1}$.

Remark. Under the neutrality hypothesis, the number of living individuals $N^i(t)$ of the species i have the same probability distribution at time $t \geq 0$, for $i = 1, \ldots, S$

and these variables are independent. So that, let denote by $N(t)$ any of the above variables. Since $0 \leq Z_J(t) = N(tJ)/J \leq 1$ for all $t \geq 0$, the sequence $(Z_J(t))_{J\in\mathbb{N}}$ is uniformly integrable for all t. Therefore, the convergence in distribution of Z_J to Z yields

$$\lim_{J\to\infty}\left[\mathbb{E}\left(\frac{N(tJ)}{J}\right)\right] = \mathbb{E}(Z(t)) = \int_0^1 x\rho_t(x)dx, \tag{S13}$$

where ρ_t is the solution to the Master Equation. Also, under the Neutrality Hypothesis one has the following approach to compute the probability of finding a species with n individuals at time tJ.

$$\begin{aligned} p_{n,J} &= \mathbb{P}(N_J(tJ) = n) \\ &= \mathbb{P}(n \leq N_J(tJ) < n+1) \\ &= \mathbb{P}\left(\frac{n}{J} \leq \frac{N_J(tJ)}{J} < \frac{n+1}{J}\right) \\ &= \mathbb{P}\left(\frac{n}{J} \leq Z_J(t) < \frac{n+1}{J}\right). \end{aligned} \tag{S14}$$

For J large enough, $\mathbb{P}\left(\frac{n}{J} \leq Z_J(t) < \frac{n+1}{J}\right)$ is approached by $\mathbb{P}\left(\frac{n}{J} \leq Z(t) < \frac{n+1}{J}\right)$, and then letting $t \to \infty$, the above expression becomes equivalent to

$$\int_{\frac{n}{J}}^{\frac{n+1}{J}} \frac{1}{B(\alpha, \alpha(S-1))} x^{\alpha-1}(1-x)^{\alpha(S-1)-1}dx. \tag{S15}$$

And, similarly,

$$\lim_{t\to\infty}\lim_{J\to\infty}\mathbb{E}(\frac{N_J(tJ)}{J}) = \int_0^1 \frac{1}{B(\alpha, \alpha(S-1))} x^{\alpha}(1-x)^{\alpha(S-1)-1}dx. \tag{S16}$$

Finally, as it has been the tradition in neutral theory we can derive the typical species abundance distribution (SAD), or expected number of species having n individuals in the focal community. That is, the probability of occurrence of that event is given by (S14). Since the species are independent and identical, we have a binomial distribution with parameters $(S, p_{n,J})$, so that its mean value is simply $Sp_{n,J}$. Therefore, it can be approached for J large enough by

$$S\mathbb{P}\left(\frac{n}{J} \leq Z(t) < \frac{n+1}{J}\right),$$

and letting $t \to \infty$ this is asymptotically equivalent to

$$S\int_{\frac{n}{J}}^{\frac{n+1}{J}} \frac{1}{B(\alpha, \alpha(S-1))} x^{\alpha-1}(1-x)^{\alpha(S-1)-1}dx, \tag{S17}$$

which is our approximation to the SAD $\langle\phi_n\rangle$. That is

$$\langle\phi_n\rangle \sim \frac{S}{JB(\alpha, \alpha(S-1))}\left(\frac{n}{J}\right)^{\alpha-1}\left(1-\left(\frac{n}{J}\right)\right)^{\alpha(S-1)-1} \tag{S18}$$

Table S 1: Fit of the discrete Beta distribution (eqn. 28) to fifteen plant and animal communities. Data for communities 1-6 comes from (*5*), 7-9 from (*6*) 10 from (*7*), 11-12 from (*8*) and 13-15 from (*9*). The estimation of α and β was done by optimisation based on the Nelder-Mead method implemented in the maximum likelihood function mle2, included in library bbmle for R. For each community, the Volkov model was simulated using function volkov included in library untb for R. Observed richness (S) and total abundance (J) were directly calculated from data and passed to the function as arguments. On the other hand, parameters theta and m required by this function were estimated using software *tetame* (*10, 11*). Comparison between observed and predicted frequency distribution were done using Pearson's correlation (P).

	Community	S	J	α	β	P_{beta}	P_{Volkov}
1	Sinharaja	167	16936	0.2498	41.4681	0.915	0.931
2	Pasoh	678	26554	0.3868	261.8361	0.978	0.980
3	Korup	308	24591	0.2783	85.4508	0.945	0.947
4	Yasuni	821	17546	0.4872	399.4592	0.967	0.967
5	Lambir	1004	33175	0.4290	430.3299	0.987	0.988
6	Barro Colorado Island	225	21457	0.2773	62.1195	0.897	0.897
7	Hangklip	247	23756	0.2538	62.4335	0.927	0.361
8	Cederberg	247	11561	0.3025	74.4123	0.849	0.899
9	Zuurberg	114	8806	0.3709	41.9154	0.415	0.409
10	Terborgh	245	1663	0.9877	493.8225	0.854	0.948
11	Fisher Butterflies	501	3306	0.9877	493.8225	0.891	0.986
12	Fisher Lepidoptera	180	2020	0.6976	124.8675	0.905	0.950
13	Dornelas Indo Pacific	450	3779	0.6427	288.5521	0.840	0.903
14	Dornelas Papua New Guinea	403	2520	0.8557	344.0041	0.864	0.939
15	Dornelas Solomon Islands	268	1201	1.1268	300.8495	0.834	0.940

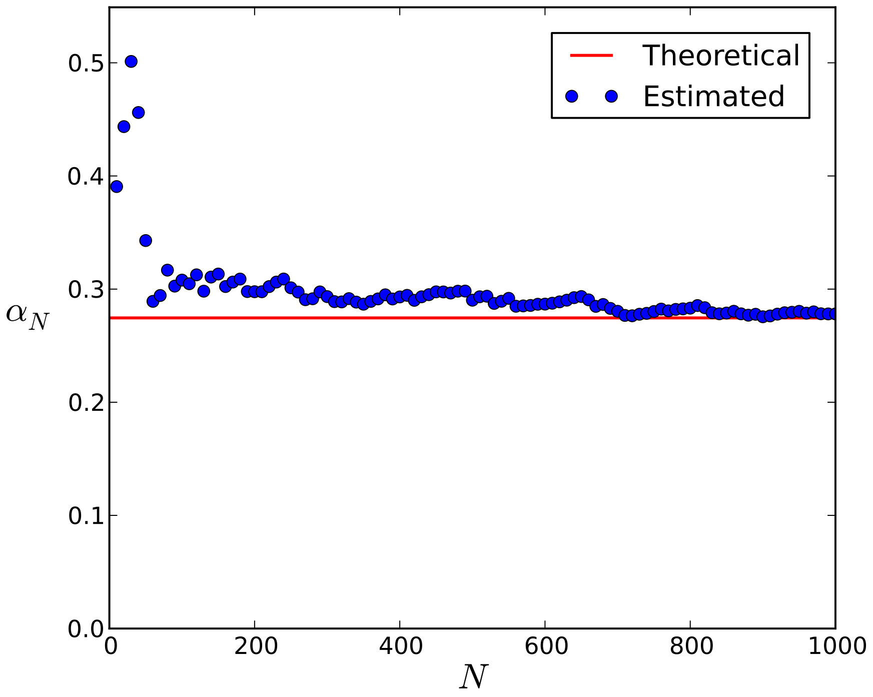

Figure S 1: Estimation of the alpha parameter of the Beta distribution Eq. (27) in the main text, using different number of trajectories of the stochastic process $Z(t)$ from Eq.(24) in the main text

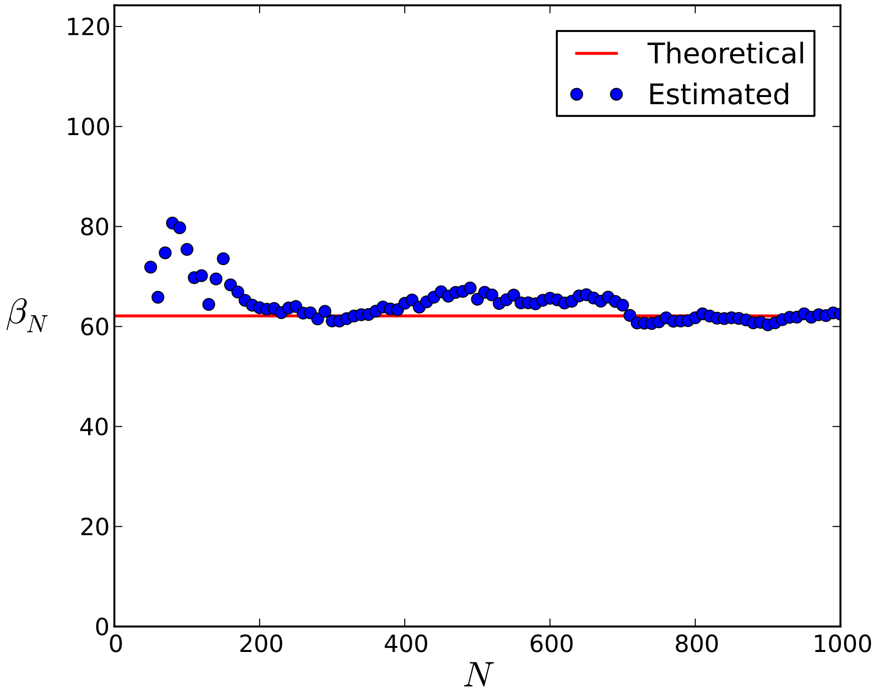

Figure S 2: Estimation of the beta parameter of the Beta distribution Eq. (27) in the main text, using different number of trajectories of the stochastic process $Z(t)$ from Eq.(24) in the main text

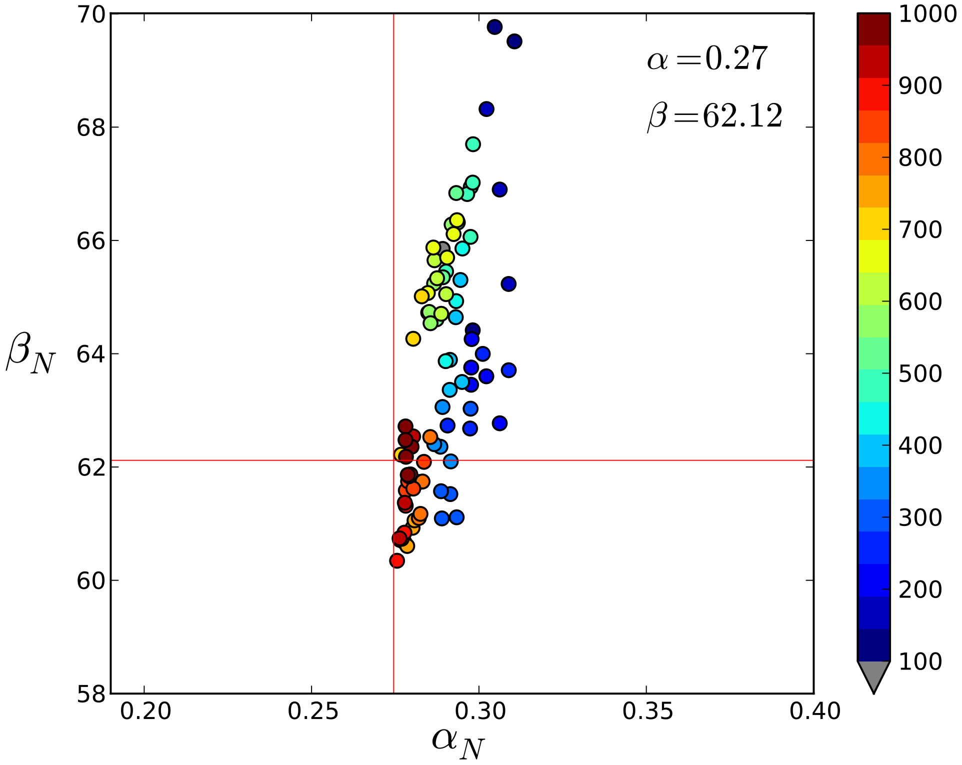

Figure S 3: Bivariate representation of the estimated values of α and β using different number of trajectories as explained in Figures S1,2

References

1. Brémaud P (1981) *Point processes and queues: martingale dynamics.* (Springer-Verlag, New York).

2. Rebolledo R (1979) La méthode des martingales appliquée à l'étude de la convergence en loi de processus. *Bulletin of the SMF. Mémoires*, 62:129p.

3. Rebolledo R (1980) Sur l'existence de solutions à certains problèmes de semimartingales. *C. R. Acad. Sci. Paris Sér. A-B*, 290:A843-A846.

4. Karatzas I, Shreve S (2012) *Brownian motion and stochastic calculus.* Springer Science and Business Media.

5. Volkov I, Banavar JR, He F, Hubbell SP, Maritan A (2005) Density dependence explains tree species abundance and diversity in tropical forests. *Nature* 438: 658-661.

6. Latimer AM, Silander JA, Cowling RM (2005) Neutral ecological theory reveals isolation and rapid speciation in a biodiversity hot spot. *Science* 309: 1722-1725.

7. Terborgh J, Robinson SK, Parker III TA, Munn CA, Pierpont N (1990) Structure and organization of an Amazonian forest bird community. *Ecological Monographs* 60: 213-238.

8. Fisher RA, Corbet AS, Williams CB (1943) The relation between the number of species and the number of individuals in a random sample of an animal population. *J Anim Ecol* 12: 42-58.

9. Dornelas M, Connolly SR, Hughes TP (2006) Coral reef diversity refutes the neutral theory of biodiversity. *Nature* 440: 80-82.

10. Chave J, Alonso D, Etienne, RS. (2006). Comparing models of species abundance. *Nature* 441: E1.

11. Jabot F, Etienne, RS, Chave J. (2008). Reconciling neutral community models and environmental filtering: theory and an empirical test. *Oikos* 117: 1308-132.
